# Quantitative Bias Analysis for a Misclassified Confounder: A Comparison Between Marginal Structural Models and Conditional Models for Point Treatments

**DOI:** 10.1097/EDE.0000000000001239

**Published:** 2020-08-05

**Authors:** Linda Nab, Rolf H. H. Groenwold, Maarten van Smeden, Ruth H. Keogh

**Affiliations:** From the a Department of Clinical Epidemiology, Leiden University Medical Center, Leiden, The Netherlands; b Department of Biomedical Data Sciences, Leiden University Medical Center, Leiden, The Netherlands; c Department of Medical Statistics, London School of Hygiene and Tropical Medicine, London, United Kingdom.

**Keywords:** Inverse probability weighting, Marginal structural models, Misclassification, Point-treatment study, Quantitative bias analysis

## Abstract

Observational data are increasingly used with the aim of estimating causal effects of treatments, through careful control for confounding. Marginal structural models estimated using inverse probability weighting (MSMs-IPW), like other methods to control for confounding, assume that confounding variables are measured without error. The average treatment effect in an MSM-IPW may however be biased when a confounding variable is error prone. Using the potential outcome framework, we derive expressions for the bias due to confounder misclassification in analyses that aim to estimate the average treatment effect using an marginal structural model estimated using inverse probability weighting (MSM-IPW). We compare this bias with the bias due to confounder misclassification in analyses based on a conditional regression model. Focus is on a point-treatment study with a continuous outcome. Compared with bias in the average treatment effect in a conditional model, the bias in an MSM-IPW can be different in magnitude but is equal in sign. Also, we use a simulation study to investigate the finite sample performance of MSM-IPW and conditional models when a confounding variable is misclassified. Simulation results indicate that confidence intervals of the treatment effect obtained from MSM-IPW are generally wider, and coverage of the true treatment effect is higher compared with a conditional model, ranging from overcoverage if there is no confounder misclassification to undercoverage when there is confounder misclassification. Further, we illustrate in a study of blood pressure-lowering therapy, how the bias expressions can be used to inform a quantitative bias analysis to study the impact of confounder misclassification, supported by an online tool.

## Abstract 2

Supplemental Digital Content is available in the text.

The aim of many observational epidemiologic studies is to estimate a causal relation between an exposure and an outcome, through careful control for confounding. In the case of a point-treatment, that is, estimating the effect of a treatment at a single time point on a subsequent outcome, many methods exist that aim to estimate average treatment effects. These include traditional conditional regression analysis and marginal structural models estimated using inverse probability weighting (MSMs-IPW).^1,2^ Unlike conditional regression, MSMs extend to estimation of joint treatment effects over multiple time points in longitudinal settings with time-dependent confounding.^1,3^

To obtain valid inference, MSMs-IPW, like other methods to control for confounding, assume that confounding variables are measured without error, an assumption hardly ever warranted in observational epidemiologic research.^4–7^ A type of measurement error is classification error, which occurs when categorical variables are misclassified. For instance, smoking status (smoker versus nonsmoker) is prone to classification error but has been used as a confounding variable in studies investigating dialysis on mortality^8^ and iron supplement use during pregnancy on anemia at delivery.^9^ Another example of the use of a potentially misclassified confounding variable is alcohol use during pregnancy (yes versus no) in studies investigating associations between exposure to triptans during fetal life and risk of externalizing and internalizing behaviors in children.^10^ In all aforementioned examples, MSMs were used to estimate the exposure–outcome relation, but the assumption of error-free confounding variables is possibly violated and may lead to bias in the treatment effect estimator.

There is a substantial literature on bias due to measurement error in confounding variables in conditional regression analyses,^11–15^ but the impact of measurement error in confounding variables in causal inference methods, such as MSMs-IPW, has not received much attention. One exception is a study by Regier et al^16^ that showed by means of a simulation study that measurement error in continuous confounding variables can introduce bias in the average treatment effect estimator in a point-treatment study. McCaffrey et al^17^ proposed a weighting method to restore the treatment effect estimator when covariates are measured with error.

We provide a discussion of measurement error in a confounding variable. In addition, we derive expressions that quantify the bias in the average treatment effect estimator if a dichotomous confounding variable is misclassified, focusing on a point-treatment study with a continuous outcome. These expressions allow us (1) to quantify the bias due to classification error in a confounding variable in MSMs-IPW and to compare this with the bias from a conditional regression analysis and (2) to inform quantitative bias analyses.^18–20^ We use simulation results to study the finite sample performance of an marginal structural model estimated using inverse probability weighting (MSM-IPW) compared with that of conditional regression models if classification error in a confounding variable is present. We illustrate our quantitative bias analysis in a study of the effect of blood pressure-lowering drugs on blood pressure.

## SETTINGS AND IMPACT OF MEASUREMENT ERROR, NOTATION, AND ASSUMPTIONS

Let 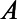 denote the treatment indicator and 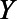 the outcome. Let there be a variable 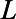 that confounds the association between treatment and outcome and suppose that, instead of confounding variable 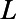, the error-prone confounding variable 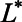 is observed. We consider two settings in which measurement error in confounding variables may occur and discuss the impact of measurement error in both settings.

### Settings and Impact of Measurement Error

**FIGURE 1.**
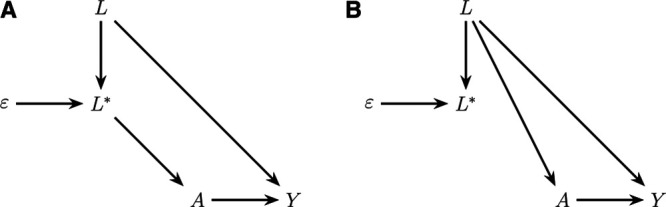
Measurement error 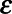 in variable 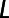 that confounds the association between treatment 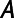 and outcome 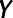 in two settings illustrated in directed acyclic graphs. A, Setting 1: Treatment 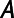 is initiated based on the error-prone confounding variable 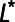. B, Setting 2: Treatment 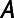 is initiated based on confounding variable 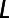.

The directed acyclic graph (DAG) in Figure 1 illustrates setting 1. In this setting, treatment initiation is based on the error-prone confounding variable. Consider, for example, a study investigating the relation between the use of antidepressant drugs (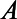) and the risk of a hip fracture (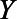).^21^ Benzodiazepine use may be a confounding variable but is prone to classification error because only prescription information may be available and over-the-counter use is often unknown. The clinician initiating the antidepressant drugs might not know their patient’s over-the-counter use and initiates treatment based on the observed error-prone benzodiazepine use (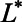) instead of actual use (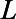), as depicted in Figure 1A. Here, conditioning on the error-prone L* will block the backdoor path from treatment 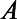 to outcome 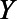. Thus, it is sufficient to control for the error-prone confounding variable to estimate the causal effect of treatment on outcome. This means that measurement error in a confounding variable will not always lead to bias.

The DAG in Figure 1B illustrates setting 2, in which treatment initiation is based on 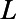, but only a proxy of 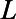 is observed (L*). An example here might be a study investigating the effect of influenza vaccination (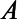) on mortality (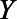) in the elderly population.^22^ Frailty (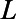) possibly confounds the association between influenza vaccination and mortality. Frailty is observed by a clinician, but only a proxy of frailty (L*) may be available in electronic health records, as depicted in Figure 1B. Here, conditioning on L* will not fully adjust for confounding by 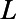, because conditioning on L* does not block the backdoor path from 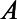 to 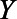 via 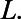


### Notation and Assumptions

We will now continue investigating the impact of classification error in setting 2, by focusing on the setting where 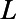 is a dichotomous confounding variable and 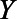 a continuous outcome. We use the potential outcomes framework.^23,24^ Let 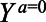 denote the outcome that an individual would have had if treatment 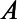 was set to 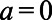, and let 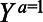 denote the outcome if treatment 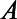 was set to 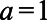. We assume that L* is nondifferentially misclassified with respect to the outcome (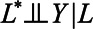) and to the treatment (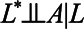). Let 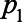 denote the sensitivity of L* and 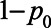, the specificity of L* (i.e., 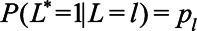). We also denote the probability of treatment given the level of 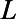 by 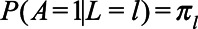 and the prevalence of 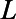 by 
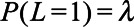
. Here, we assume that 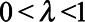 because we are not interested in populations where 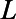 is present or absent in everyone. Finally, we assume no measurement error in exposure and outcome.

We also assume that the following causal assumptions are satisfied to recover the causal effect of treatment on the outcome. Under the consistency assumption, we require that we observe 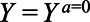 if the individual is not exposed or 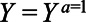 if the individual is exposed.^25^ Further, we assume that the potential outcome 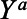 for an individual does not depend on treatments received by other individuals and that there are not multiple versions of treatment, also referred to as Stable Unit Treatment Value Assumption.^26^ Additionally, we assume conditional exchangeability, i.e., given any level of 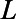, if the untreated group had in fact received treatment, then their expected outcome would have been the same as that in the treated, and vice versa.^25^ In notation, 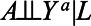, for 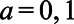. Finally, we assume 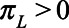 for 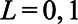 (positivity).^27^

For causal contrasts, we compare expected potential outcomes (i.e., counterfactual outcomes) under the two different treatments. The average causal effect of the treatment on the outcome is 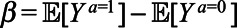. Under the above defined assumptions, the conditional effect of treatment 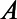 on outcome 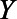 can be defined through the following linear model:


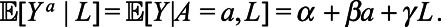
(1)

Estimates for 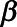 in the above model can be obtained by fitting a conditional regression model. Alternatively, the effect of treatment 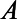 on outcome 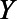 may be estimated by fitting an MSM:


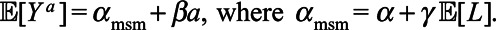
(2)

Estimates for 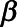 in the above model can be obtained by IPW estimation: by fitting a linear regression model for 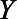 on 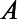 where the contribution of each individual is weighted by 1 over the probability of that individual’s observed treatment given 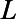, estimating the marginal treatment effect.^2^ Because our focus is on linear models and we make the simplifying assumption that the effect of 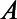 on 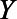 does not vary between strata of 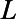, the conditional and marginal treatment effects, denoted by 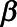 in model equations 1 and 2, respectively, are equal. This is not generally true for nonlinear models due to noncollapsibility.^2^ We assume that the effect of 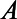 on 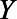 does not vary between strata of 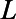, to derive bias expressions that are easier to use in practice and require fewer parameters.^28^

## QUANTIFICATION OF BIAS DUE TO CLASSIFICATION ERROR IN A CONFOUNDING VARIABLE

Our aim is to study the effect of using the misclassified confounding variable L* in place of the confounding variable 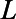 in the conditional regression model or in the model for the weights used to fit the MSM on the average treatment effect estimator in the setting where 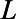, not L*, influences treatment initiation (setting 2 above).

### Conditional Model

By the law of total expectation, the expected value of the outcome 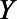 given treatment 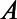 and L* is (see eAppendix 1; http://links.lww.com/EDE/B698 section 1 for further detail),


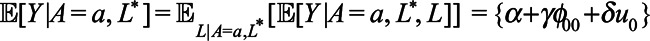



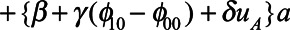



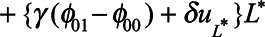


where 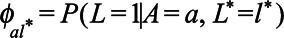, 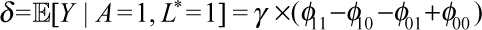 and 
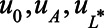
 represent the coefficients of the linear model 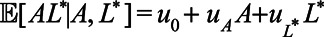, modeling the mean of 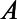 times L* (i.e., 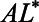) given 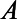 and L* (see next paragraph for an explanation of why these appear). The coefficient for treatment 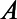 in the above model is 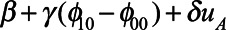, and is therefore biased for the parameter of interest (i.e., 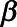). By rewriting 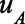 in terms of 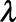, 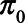, 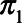, 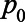 and 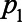 (see eAppendix 1 section 1), we find that the bias due to classification error in L* in the average treatment effect in a conditional regression model is as follows:


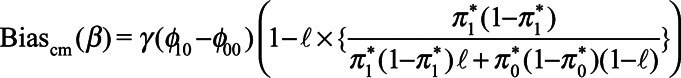



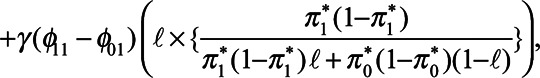
(3)

where 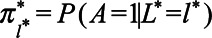, 
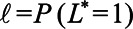
 (see eAppendix 1 section 1 for a derivation).

We focused on a model for 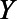 conditional on 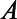 and L* which includes only main effects of 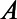 and L*, as this is typically done in practice when replacing 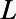 with L*. In fact, it can be shown that when the model for 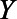 given 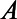 and 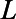 includes only main effects of 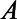 and 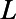, the implied correctly specified model for 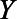 given 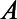 and L* also includes an interaction between 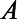 and L*, explaining the appearance of 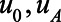, and 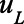 in the above because the interaction is not modeled. See eAppendix 1 section 1 for the bias in case an interaction is modeled

### Marginal Structural Model Estimated Using Inverse Probability Weighting

An MSM-IPW proceeds by fitting a linear regression for outcome 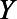 on treatment 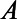, where the contribution of each individual is weighted by 1 over the probability of that individual’s observed treatment given misclassified L*.^2^ An estimator for the average treatment effect 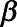 is as follows:


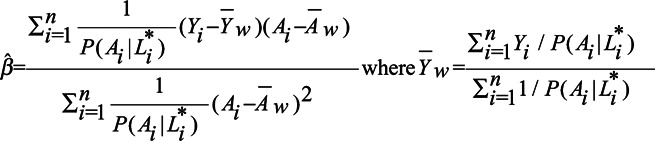



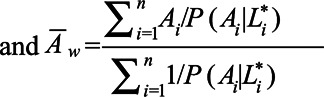


It can be shown that 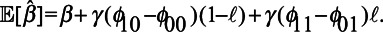 Consequently, the bias in the average treatment effect in an MSM-IPW is as follows:


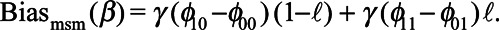
(4)

We refer to eAppendix 1 section 2 for a derivation of the above formula.

### Exploration of Bias

To study the bias due to misclassification from the conditional model and MSM-IPW, we explore bias expressions (equations 3 and 4).

#### Null Bias

To confirm the derived bias expressions, we consider three trivial conditions where bias in the average treatment effect estimator is expected to be null, in line with general understanding of causal inference.^29^ (1) If there is no classification error in L*, i.e., specificity is 1 (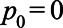) and sensitivity is 1 (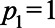), it follows that 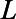 corresponds to L*, irrespective of treatment level (i.e., 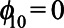, 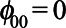, 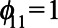, and 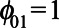). (2) If the true relation between 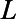 and 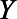 is null (i.e., 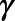 is zero, thus there is no arrow from 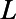 to 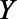 in Figure 1B). (3) If 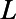 does not affect the probability of receiving treatment (i.e., 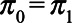, thus there is no arrow from 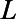 to 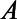 in Figure 1B), the probability that 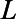 is 1 depends on the value of L* but no longer on 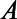 (i.e., 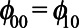 and 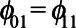). Bias is null under these conditions for both models (MSM-IPW and conditional model). Because the bias expressions are strictly monotonic, the bias in an MSM-IPW cannot be negative if the bias in the conditional model is positive and vice versa (i.e., the bias will be in the same direction for both models).

**FIGURE 2.**
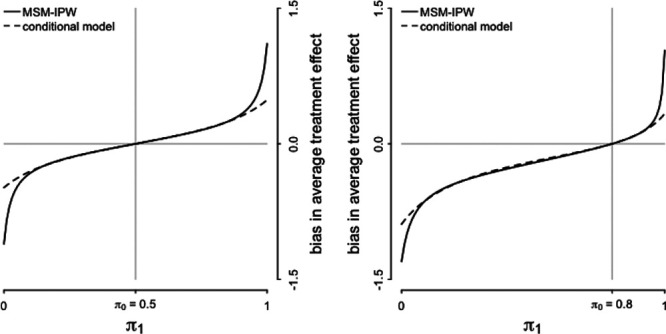
Visualization of the direction and magnitude of the bias in the average treatment effect estimator in relation to the prevalence of treatment among individuals with the confounding variable present. In this visualization, the confounding variable 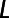 is misclassified with a sensitivity of 0.9 and specificity of 0.95. Consequently, the average treatment effect estimated in an MSM-IPW or conditional regression model is biased, independent of true average treatment effect. The prevalence of 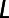 is 50% (i.e., 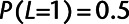). The direction and magnitude of the bias depend on (1) the strength and direction of the association between 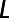 and treatment (denoted by 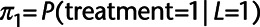 and 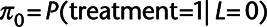, here set at 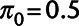 in the left-hand-side plot and 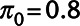 in the right-hand-side plot); and (2) the strength and direction of the association between 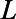 and the outcome (denoted by 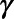 in the text and here set at 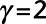). Larger values of 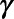 will result in steeper curves; 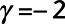 will mirror the graph in 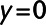.

#### Equal Biases

The bias in the average treatment effect in a conditional regression analysis is equal to that in an MSM-IPW if bias in both models is null (see above). We also see that bias expressions (equations 3 and 4) show that bias for the two methods is equal if the term between curly brackets in equation 3 is equal to 1, which is the case if (1) 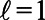; (2) 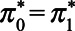; and (3) 
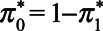
. If conditions (1) and/or (2) are met, there is no bias in an MSM-IPW nor in a conditional model. Under condition (3), bias is generally non-null (except if, for example, 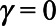, see Null Bias).

#### Sign and Magnitude of Bias

**FIGURE 3.**
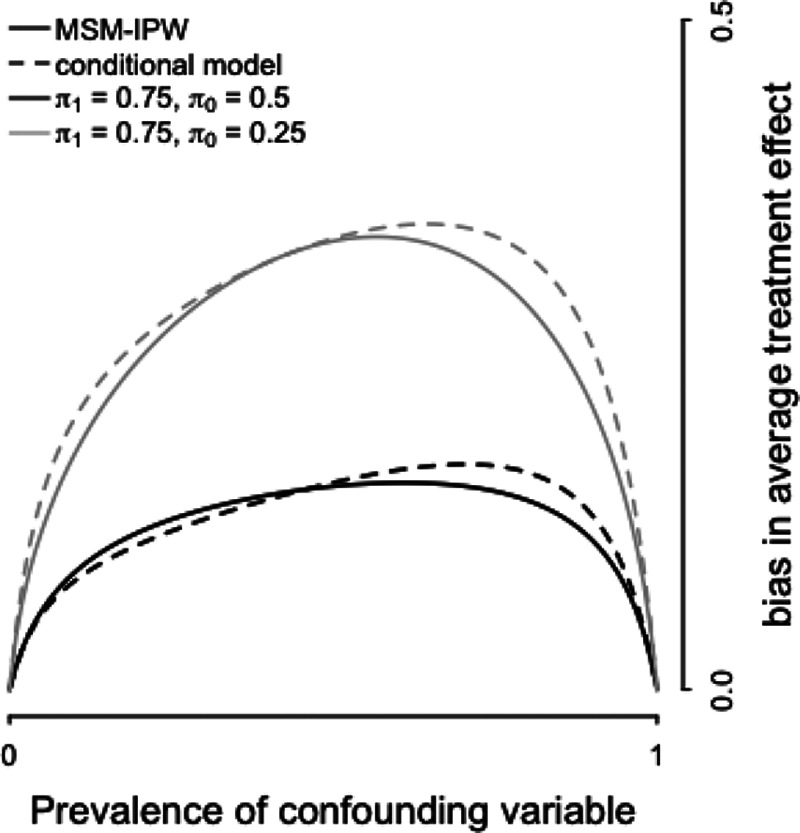
Visualization of the magnitude of the bias in the average treatment effect estimator in relation to the prevalence of a confounding variable. In this visualization, the confounding variable 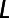 is misclassified with a sensitivity of 0.9 and specificity of 0.95. Consequently, the average treatment effect estimated in an MSM-IPW or conditional regression model is biased, independent of true average treatment effect. The confounding variable is positively associated with treatment (i.e., here 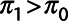, where 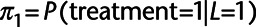 and 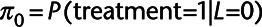), and outcome (denoted by 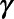 in the text and here set at 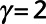). The magnitude of the bias depends on the prevalence of the confounding variable (i.e., 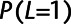). Larger values of 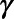 will result in steeper curves.

**FIGURE 4.**
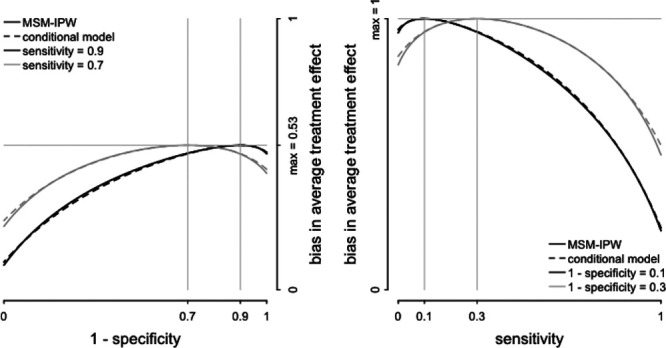
Visualization of the magnitude of the bias in the average treatment effect estimator in relation to specificity and sensitivity of a misclassified confounding variable. In this visualization, the prevalence of the confounding variable 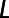 is 50% (i.e., 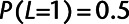), the association between 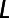 and treatment (denoted by 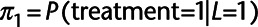 and 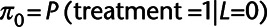) and outcome is positive (denoted by 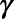 in the text and here set at 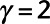). Given these values, if 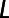 is misclassified, the average treatment effect estimated in an MSM-IPW or conditional regression model is biased, independent of true average treatment effect. The magnitude of the bias depends on the specificity and sensitivity of 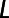 and is maximal if sensitivity equals 1 − specificity. The strength of the association between 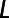 and treatment is greater in the right-hand-side plot (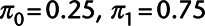) compared with the left-hand-side plot (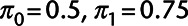), and consequently, bias is greater. Larger values of 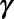 will result in steeper curves.

Figures 2–4 illustrate the contributions to bias in the average treatment effect due to misclassification components (sensitivity and specificity) and due to confounding components (prevalence of confounding variable, strength of association between confounding variable and treatment and outcome) in a conditional model and an MSM-IPW, obtained by using the bias expressions.

Figure 2 shows that (1) the bias is positive if both the association between 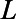 and treatment and 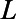 and outcome are positive (i.e., 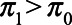 and 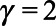, respectively) and (2) the bias is greater if the difference between 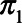 and 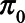 is greater (i.e., if the strength of the association between 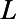 and treatment is greater). In contrast, the bias is negative if 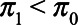, whereas 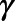 is positive. In case 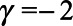, Figure 2 is mirrored in 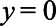, and consequently, bias is negative if 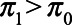 and positive if 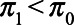. An increment in 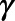 will result in greater bias and steeper curves in Figure 2. Figure 3 shows that the magnitude of the bias depends on the prevalence of 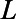. Further, it shows that bias is greater if the strength of association between 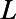 and treatment is greater. Figure 4 shows that, generally, the bias is greater if L* has lower specificity and sensitivity. Moreover, for a fixed sensitivity, bias is minimal if specificity equals 1 and is maximal if 1 − specificity equals sensitivity; by fixing specificity, bias is minimal if sensitivity equals 1 and is maximal if sensitivity equals 1 − specificity. Figure 4 shows that the bias is greater if the strength of the association between 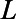 and treatment is greater. An increment in 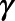 will result in greater bias and steeper curves in Figure 4. An online application can be used to obtain bias plots for other combinations of the parameters available at https://lindanab.shinyapps.io/SensitivityAnalysis.

### Simulation Study

We conducted a simulation study to study the finite sample properties of MSMs estimated using IPW and conditional models if there is classification error in the confounding variable. Five thousand data sets were generated with sample sizes of 1,000 and 100, using the following data-generating mechanisms:


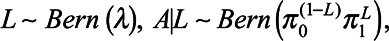



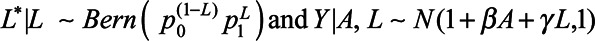


We studied five different scenarios, of which the parameters values can be found in Table 1. In all scenarios, the average treatment effect 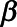 (estimand) is 1 and the association between the confounding variable 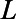 and outcome 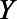 is 2 (i.e., 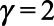). In scenario 0, we assume no classification error. In scenarios 1–4, we assume that L* has a specificity of 0.95 (i.e., 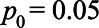) and a sensitivity of 0.90 (i.e., 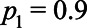). In scenario 1, bias in the average treatment effect estimator is expected to be negative because the probability of receiving treatment given that 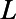 is not present is greater than receiving treatment given that 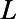 is present, and the association between 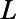 and 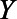 is positive (i.e., 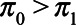 and 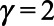). In contrast, in scenarios 2 and 3, bias in the average treatment effect estimator is expected to be positive, because 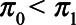 and 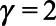. Further, after investigation of Figure 3, we expect that bias in the average treatment effect estimated in a conditional model is greater than that in an MSM-IPW in scenarios 2 and 3. Finally, in scenario 4, we expect that bias in the average treatment effect from the conditional model is equal to that in an MSM-IPW.

**TABLE 1.** Values of the Parameters in the Five Different Simulation Scenarios

Scenario	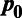	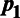	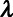	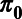	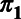	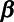	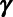
0	0	1	0.50	0.50	0.75	1	2
1	0.05	0.90	0.50	0.90	0.45	1	2
2	0.05	0.90	0.80	0.25	0.75	1	2
3	0.05	0.90	0.80	0.50	0.75	1	2
4	0.05	0.90	0.45	0.50	0.75	1	2

#### Model Estimation and Performance Measures

We obtained the average treatment effect 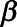 (estimand) by fitting a conditional model using conditional regression and by fitting an MSM-IPW, both using the misclassified L* instead of 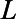 from the data-generating mechanism. For the MSM-IPW analysis, we used the R package ipw.^30,31^ Performance of both models was evaluated in terms of the bias, the mean squared error of the estimated treatment effect (MSE), the percentages of 95% confidence intervals that contain the true value of the estimand (coverage), the empirical standard deviation of the estimated treatment effects (empSE), and mean model-based standard error of the estimated treatment effect. We estimated robust model-based standard errors of the average treatment effect in an MSM-IPW using the R package survey.^32^ We calculated Monte Carlo standard errors for all performance measures,^33^ using the R package rsimsum.^34^ Additionally, we calculated the theoretical bias of the average treatment effect in both methods based on the bias expressions (equations 3 and 4).

## RESULTS

Table 2 shows the results of the simulation study. Bias found in the simulation study corresponds to the theoretical bias derived from the bias expressions. The empirical standard deviation of the average treatment effect estimates (empSE) from the MSM-IPW is equal to or greater than that from the conditional model. Yet, in the scenarios where bias in the average treatment effect in the MSM-IPW was smaller than bias in the conditional model (scenarios 2 and 3), empSE of both methods was equal, and hence, MSE is smaller for one method if also bias is smaller. Furthermore, the (robust) model-based standard errors of the average treatment effect in an MSM-IPW are conservative and greater than the empirical standard errors, because the uncertainty in estimating the treatment weights is not taken into account. Allowing for the estimation of the weights will shrink the standard errors.^2,35^ We chose not to use a less conservative standard error estimation for MSM-IPW, such as bootstrapping, because our goal was to frame this simulation as investigating the properties of the commonly used MSM-IPW estimation procedure. Consequently, confidence intervals of the treatment effect obtained in an MSM-IPW are generally wider and coverage of the true treatment effect is higher compared with a conditional model, ranging from overcoverage if there is no classification error to smaller undercoverage when there is classification error.

**TABLE 2.** Results of Simulation Study Studying the Finite Sample Properties of a marginal structural model estimated using inverse probability weighting (MSM-IPW) and a CM If There Is Classification Error in the Confounding Variable

Method	Sample Size	Scenario^a^	Bias (Formula)^b^	Bias	MSE^c^	Coverage	empSE^d^	modelSE^e^
MSM-IPW	1,000	0	0.00	0.00 (0.001)	0.00 (0.000)	0.99 (0.001)	0.07 (0.001)	0.10 (0.000)
		1	−0.42	−0.42 (0.001)	0.18 (0.001)	0.03 (0.002)	0.10 (0.001)	0.11 (0.000)
		2	0.14	0.14 (0.001)	0.03 (0.000)	0.67 (0.007)	0.08 (0.001)	0.09 (0.000)
		3	0.29	0.29 (0.001)	0.09 (0.001)	0.08 (0.004)	0.08 (0.001)	0.09 (0.000)
		4	0.15	0.15 (0.001)	0.03 (0.000)	0.68 (0.007)	0.08 (0.001)	0.10 (0.000)
	100	0	0.00	0.00 (0.003)	0.05 (0.001)	0.99 (0.001)	0.22 (0.002)	0.31 (0.000)
		1	−0.42	−0.42 (0.005)	0.29 (0.005)	0.78 (0.006)	0.34 (0.003)	0.37 (0.001)
		2	0.14	0.14 (0.004)	0.08 (0.002)	0.94 (0.003)	0.25 (0.003)	0.29 (0.000)
		3	0.29	0.29 (0.004)	0.15 (0.002)	0.84 (0.005)	0.26 (0.003)	0.28 (0.000)
		4	0.15	0.15 (0.004)	0.08 (0.002)	0.95 (0.003)	0.25 (0.002)	0.31 (0.000)
CM	1,000	0	0.00	0.00 (0.001)	0.00 (0.000)	0.95 (0.003)	0.07 (0.001)	0.07 (0.000)
		1	−0.34	−0.34 (0.001)	0.12 (0.001)	0.02 (0.002)	0.09 (0.001)	0.08 (0.000)
		2	0.16	0.16 (0.001)	0.03 (0.000)	0.46 (0.007)	0.08 (0.001)	0.08 (0.000)
		3	0.32	0.32 (0.001)	0.11 (0.001)	0.02 (0.002)	0.08 (0.001)	0.08 (0.000)
		4	0.15	0.15 (0.001)	0.03 (0.000)	0.49 (0.007)	0.08 (0.001)	0.07 (0.000)
	100	0	0.00	0.00 (0.003)	0.05 (0.001)	0.95 (0.003)	0.22 (0.002)	0.22 (0.000)
		1	−0.34	−0.33 (0.004)	0.19 (0.003)	0.73 (0.006)	0.29 (0.003)	0.27 (0.000)
		2	0.16	0.16 (0.004)	0.09 (0.002)	0.90 (0.004)	0.25 (0.003)	0.25 (0.000)
		3	0.32	0.32 (0.004)	0.17 (0.003)	0.74 (0.006)	0.26 (0.003)	0.25 (0.000)
		4	0.15	0.15 (0.003)	0.08 (0.002)	0.90 (0.004)	0.24 (0.002)	0.24 (0.000)

a In all scenarios, the average treatment effect (estimand) is 1 (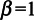) and the effect of the confounding variable on the outcome is 2 (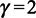). Five thousand data sets were generated. Monte Carlo standard errors are shown between brackets. In scenario 0, there is no classification error (specificity and sensitivity of the misclassified confounding variable are 1, i.e., 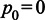 and 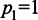). In scenarios 1–4, the specificity of the misclassified confounding variable is 0.95 (i.e., 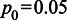) and the sensitivity is 0.9 (i.e., 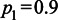). The prevalence of the confounding variable (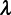) and the probability of receiving treatment if the confounding is not present or present (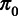 and 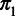, respectively) are set as follows in the scenarios: scenario 0: 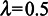, 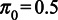, 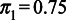; scenario 1: 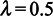, 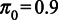, 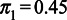; scenario 2: 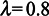, 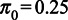, 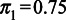; scenario 3: 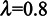, 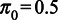, 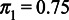; and scenario 4: 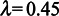, 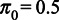, 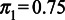.

b Bias based on bias expressions (equations 3 and 4) in the text.

c Mean squared error.

d Empirical standard error.

e Model-based standard error.

## ILLUSTRATION: QUANTITATIVE BIAS ANALYSIS OF CLASSIFICATION ERROR IN A CONFOUNDING VARIABLE

Quantitative bias analysis provides a tool to incorporate uncertainty in study results due to systematic errors.^18,20^ Using an example study of blood pressure-lowering therapy, we illustrate how the bias expressions (equations 3 and 4) can be used to perform a quantitative bias analysis for misclassification of a confounding variable.

### Application

For our illustration we use data of the National Health And Nutritional Examination Survey (NHANES),^36,37^ more details can be found in the Supplement 2; http://links.lww.com/EDE/B698. Specifically, we study the effect of diuretic use (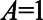) in comparison to beta blocker use 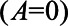 on systolic blood pressure (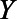) using two approaches: by inverse weighting with the propensity for diuretic or beta blocker use given self-reported categorical body mass index (BMI) (L*) and using a conditional linear regression with adjustment for self-reported categorical BMI. For this illustration, we categorize self-reported BMI into two distinct categories: underweight/normal weight (BMI 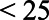 (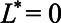)) and overweight/obese (BMI ≥25 (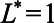)). However, we stress that one should preferably not categorize BMI in most practical applications.^38^ Moreover, we assume that dichotomizing self-reported BMI does not introduce differential misclassification.^7^

We assume that blood pressure-lowering therapy is initiated based on the true BMI (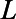) instead of the observed self-reported BMI (setting 2, Figure 1B). Further, we consider BMI the only confounding variable, and treatment and outcome to be measured without error, which is a simplification of reality. Additionally, we assume that the classification error in self-reported BMI category is nondifferential for the subject’s treatment or blood pressure (given true BMI category). Expert knowledge is needed to inform this assumption. To quantify how large the bias in the average treatment effect estimator is expected to be due to classification error in self-reported BMI category, we perform a quantitative bias analysis using the bias expressions (equations 3 and 4).

### Average Treatment Effect

Table 3 shows the average treatment effect of diuretics use in comparison to beta blocker use on mean systolic blood pressure. In an MSM-IPW, we estimated an average treatment effect (95% confidence interval [CI]) of −3.52 (−1.21, −5.74). In a conditional regression model, we estimated an average treatment effect (95% CI) of −3.48 (−1.27, −5.76).

**TABLE 3.** Average Treatment Effect of Diuretics Use Compared with Beta Blocker Use on Mean Systolic Blood Pressure in NHANES^36,37^

Model	Effect Size (CI)
Unadjusted	−4.03 (−6.30, −1.76)
Marginal structural model^a^	−3.52 (−1.21, −5.74)
Conditional model^b^	−3.48 (−1.27, −5.76)

a Estimated in a marginal structural model, by inverse weighting with the propensity for diuretic or beta blocker use given self-reported categorized body mass index (BMI).

b Estimated in a conditional regression model with adjustment for self-reported categorical BMI.

### Quantitative Bias Analysis

To inform the quantitative bias analysis, we need to make assumptions on the sensitivity and specificity of the self-reported BMI and that classification errors are nondifferential with respect to blood pressure and treatment. For the purpose of this illustration, we speculate ranges for the sensitivity and specificity of self-reported BMI category of 0.90 to 0.98. In practice, these parameters should be informed by reports in the literature and/or a researcher’s expert experience. Researchers may also decide to investigate how extreme the misclassification (measured using sensitivity and specificity) would need to be to change the conclusions of their study. We refer to the Shiny application (introduced in the subsequent section) for other choices for the sensitivity and specificity of self-reported BMI category.

**FIGURE 5.**
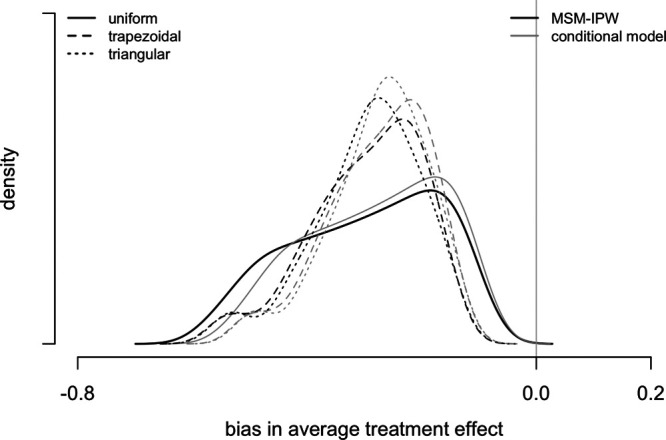
Density of predicted bias due to classification error in self-reported BMI category in NHANES.^36,37^ Bias in the average treatment effect of diuretics use compared with beta blocker use on mean systolic blood pressure by inverse weighting with the propensity for diuretic or beta blocker use given self-reported categorical BMI (MSM-IPW), and using a conditional linear regression with adjustment for self-reported categorical BMI. The specificity and sensitivity of self-reported BMI category range from 0.90 to 0.98 and are sampled from a uniform distribution, trapezoidal (with modes on one third and two third), and symmetrical triangular distribution.

By uniformly sampling from the range of plausible values of 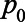 and 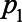 and using the bias expressions (equations 3 and 4), a distribution of possible biases is obtained (eAppendix 2; http://links.lww.com/EDE/B698 for further details). The solid line in Figure 5 shows the distribution of bias in an MSM-IPW. Mean bias is −0.31, and median bias is −0.30 (interquartile range, −0.40 to −0.20). We also considered sampling 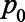 and 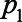 from a trapezoidal (with modes at one third and two thirds between the minimum and maximum) or a symmetrical triangular distribution. Sampling from these distributions results in mean bias approximately equal to when uniform sampling is applied, but with less spread (dashed and dotted line in Figure 5). This result suggests that the results in Table 3 are not affected much by the classification error in self-reported BMI category. In the NHANES, anthropometric measures were also taken by trained technicians. The average treatment effect when BMI measures taken by trained technicians were used instead of self-reported BMI measures is given in eAppendix 2; http://links.lww.com/EDE/B698.

## SHINY APPLICATION: AN ONLINE TOOL FOR STUDYING THE IMPACT OF A MISCLASSIFIED VARIABLE

We developed an online tool for creating bias plots (Figures 2–4) and performing quantitative bias analyses (illustrated in the previous section), available at https://lindanab.shinyapps.io/SensitivityAnalysis. The bias plots can be used to predict the implications of classification error in a confounding variable in specific study settings by varying the strength of association between the confounding variable and treatment and between the confounding variable and outcome; prevalence of the confounding variable; and specificity and sensitivity of the misclassified confounding variable. The quantitative bias analysis can be used for studying the impact of classification error in a confounding variable at the analysis stage of a study and to investigate how sensitive conclusions are to the assumption of no classification error. These bias plots can also be used to inform decisions about measurement methods or choice of variables to be extracted in the planning stage of studies.

## DISCUSSION

Inverse probability weighting and conditional models are both important and frequently used tools to adjust for confounding variables in observational studies. In this article, we derived expressions for the bias in the average treatment effect in an MSM-IPW and a conditional model. These expressions can inform quantitative bias analyses for bias due to a misclassified confounding variable.

Quantitative bias analysis of misclassified confounding variables is one example of quantitative bias analyses for observational epidemiologic studies. Several approaches exist to assess sensitivity of causal conclusions to unmeasured confounding.^28,39,40^ These aim to quantify the impact of violations of the assumption of no unmeasured confounding, although our approach aims to quantify the impact of violations of the assumption that all confounding variables are measured without error.

Several methods have been proposed to adjust for measurement error in covariates in MSMs-IPW. Pearl^41^ developed a general framework for causal inference in the presence of error-prone covariates, which yields weighted estimators in the case of a dichotomous confounding variable measured with error. The framework relies on a joint distribution of the outcome and the confounding variable. Conversely, the weighting method proposed by McCaffrey et al^17^ does not require a model for the outcome. Additionally, regression calibration,^42^ simulation-extrapolation,^43,44^ and multiple imputation^45^ have been proposed for correcting for measurement error in covariates of MSMs. These methods assume that the measurement error model is known, which may often be unrealistic. In this context, it is also important to mention previous studies of the impact of measurement error in the exposure or the end point in MSMs, which has been studied by Babanezhad et al^46^ and Shu and Yi,^47^ respectively.

If treatment is allocated based on an error-prone confounding variable, the treatment effect will not be biased (see DAG in Figure 1A). However, investigators should be careful in concluding that covariate measurement error will not affect their analysis. Suppose that there is an unmeasured variable 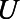 that acts as a confounding variable between the error-prone covariate L* and treatment 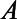. Conditioning on L* will then open a path between 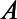 and 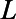 via. 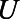 and thus confound the relation between 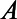 and 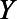.

This article considered classification error in a dichotomous confounding variable in a point-treatment study with a continuous outcome. The same principles apply to measurement error in a categorical or continuous confounding variable or when multiple confounding variables are considered, although more elaborate assumptions should then be made.^48^ Moreover, we assumed that the relation between exposure and outcome does not vary between strata of the confounding variable, i.e., that there is no treatment effect modification. Future research could extend our bias expressions by relaxing this simplifying assumption, therefore extending our results to more general settings.

MSMs-IPW are increasingly applied to longitudinal data to estimate the joint effects of treatment at multiple time points on a subsequent outcome, including time-dependent outcomes, addressing the problem of time-dependent confounding.^1,3^ There has been little work to understand or correct for the impact of misclassified or mismeasured confounding variables in this more complex setting. Our results extend directly to the time-dependent setting when the aim is to estimate the effect of a current treatment on a time-dependent outcome measured at the next time point.^49^ An area for future work is to extend our results to the setting in which the aim is to estimate the joint effects of treatment at multiple time points and to the time-dependent setting with time-varying treatments and confounding variables. An additional factor to consider in the time-varying setting is the impact of stabilized versus unstabilized weights on the bias if both numerator and denominator of the stabilized weights involve conditioning on an error-prone covariate.

The bias expressions derived in this article can be used to assess bias due to classification error in a dichotomous confounding variable. If classification error in confounding variables is suspected, a quantitative bias analysis provides an opportunity to quantitatively inform readers on the possible impact of such errors on causal conclusions.

## Supplementary Material


