# Supplementary material for: Quantitative Bias Analysis for a Misclassified Confounder: A Comparison Between Marginal Structural Models and Conditional Models for Point Treatments
Source: Epidemiology. 2020 Aug 5;31(6):796–805. doi: 10.1097/EDE.0000000000001239 (PMC7523582; doi:10.1097/EDE.0000000000001239)
Supplement: Supplementary file 1 [file ede-31-796-s001.pdf]

eAppendix1 Quantification of bias due to classification error in a confounding variable

1 Conditional model

Under the assumptions and notation described in section of the main article and by the law of total expectation, the expected value of the outcome Y given the covariates A and L^* is,

$$\begin{aligned}
\mathbb{E}[Y|A,L^*] = \mathbb{E}_{L|A,L^*}[\mathbb{E}[Y|A,L^*,L]] &= \mathbb{E}_{L|A,L^*}[\alpha + \beta A + \gamma L] \\
&= \alpha + \beta A + \gamma\, \mathbb{E}[L|A,L^*] \\
&= \alpha + \beta A + \gamma \phi_{aL^*} \\
&= \{\alpha + \gamma\phi_{00}\} + \{\beta + \gamma(\phi_{10} - \phi_{00})\}A \\
&+ \{\gamma(\phi_{01} - \phi_{00})\}L^* + \gamma(\phi_{11} - \phi_{10} - \phi_{01} + \phi_{00})AL^*,
\end{aligned}$$

which relies on the assumption that L^* is non-differentially misclassified with respect to the outcome (i.e., $L^* \perp\!\!\!\perp Y|L$) and includes an interaction between A and L^*. Further, ϕ_{al^*} is the probability that confounding variable L is one, given that treatment A is a and that misclassified confounding variable L^* is l^*, or,

$$\begin{aligned}
\phi_{al^*} &= P(L=1|A=a,L^*=l^*) \\
&= \frac{P(A|L=1,L^*=l^*)P(L=1|L^*=l^*)}{P(A=a|L^*=l^*)} \\
&= \frac{P(A=a|L=1)P(L=1|L^*=l^*)}{P(A=a|L^*=l^*)} \\
&= \frac{P(A=a|L=1)\frac{P(L^*=l^*|L=1)P(L=1)}{P(L^*=l^*)}}{P(A=a|L^*=l^*)} \\
&= \frac{P(A=a|L=1)P(L^*=l^*|L=1)P(L=1)}{P(A=a|L^*=l^*)P(L^*=l^*)} \\
&= \frac{\lambda(1-\pi_1)^{(1-a)}\pi_1^a(1-p_1)^{(1-l^*)}p_1^{l^*}}{(1-\pi^*_{l^*})^{(1-a)}\pi^{*\,a}_{l^*}(1-\ell)^{(1-l^*)}\ell^{l^*}}.
\end{aligned}$$

Here $\ell = P(L^*=l^*) = p_0(1-\lambda) + p_1\lambda$ and $\pi^*_{l^*}$ is the probability of receiving treatment A given that the misclassified confounding variable $L^* = l^*$. Note that the above is only defined if $0 < \ell < 1$ and $0 < \pi^*_{l^*} < 1$. To satisfy that $0 < \ell < 1$, we use our assumption that $0 < \lambda < 1$, and additionally, we assume that if $p_0 = 1$ then $p_1 \neq 1$, and if $p_0 = 0$ then $p_1 \neq 0$ (and vice versa). Under the assumption that $0 < \ell < 1$, it follows that,

$$\begin{aligned}
\pi^*_{l^*} &= P(A=1|L^*=l^*) \\
&= \Sigma_l P(A=1|L^*=l^*,L=l)P(L=l|L^*=l^*) \\
&= \Sigma_l P(A=1|L=l)P(L=l|L^*=l^*)
\end{aligned}$$

$$
\begin{aligned}
&= \Sigma_l P(A=1|L=l)\frac{P(L^*=l^*|L=l)P(L=l)}{P(L^*=l^*)} \\
&= \Sigma_l \pi_l \frac{(1-p_l)^{(1-l^*)}p_l^{l^*}(1-\lambda)^{1-l}\lambda^l}{(1-\ell)^{1-l^*}\ell^{l^*}},
\end{aligned}
$$

we find that $0 < \pi^*_{l^*} < 1$, if, again, $0 < \lambda < 1$, and if $p_0 = 1$ then $p_1 \neq 1$, and if $p_0 = 0$ then $p_1 \neq 0$ (and vice versa) and $0 < \pi_l < 1$ (positivity assumption).

The bias in the regression based estimator of the effect of A is $\gamma(\phi_{10}-\phi_{00})$ if the interaction between A and L^* is included in the model. However, in this model, the coefficient for A now represents the treatment effect given that L^* is null. Typically, only main effects of A and L^* are included in a regression model of Y conditional on A and L^*:

$$
\begin{aligned}
\mathbb{E}_{AL^*|A,L^*}\{\mathbb{E}[Y|A,L^*]\} &= \{\alpha+\gamma\phi_{00}\} + \{\beta+\gamma(\phi_{10}-\phi_{00})\}A + \{\gamma(\phi_{01}-\phi_{00})\}L^* \\
&+ \gamma(\phi_{11}-\phi_{10}-\phi_{01}+\phi_{00})\,\mathbb{E}[AL^*|A,L] \\
&= \{\alpha+\gamma\phi_{00}+\delta u_0\} + \{\beta+\gamma(\phi_{10}-\phi_{00})+\delta u_A\}A \\
&+ \{\gamma(\phi_{01}-\phi_{00})+\delta u_{L^*}\}L^*,
\end{aligned}
$$

where u_0, u_A, and u_{L^*} are the coefficients of the linear model $\mathbb{E}[AL^*|A,L^*] = u_0 + u_A A + u_{L^*}L^*$ and $\delta = \gamma(\phi_{11}-\phi_{10}-\phi_{01}+\phi_{00})$. Here,

$$
\begin{aligned}
u_A &= \frac{\mathbb{V}\mathrm{ar}(L^*)\,\mathbb{C}\mathrm{ov}(A,AL^*) - \mathbb{C}\mathrm{ov}(A,L^*)\,\mathbb{C}\mathrm{ov}(L^*,AL^*)}{\mathbb{V}\mathrm{ar}(L^*)\,\mathbb{V}\mathrm{ar}(A) - \mathbb{C}\mathrm{ov}(A,L^*)^2}, \\
u_{L^*} &= \frac{\mathbb{V}\mathrm{ar}(A)\,\mathbb{C}\mathrm{ov}(L^*,AL^*) - \mathbb{C}\mathrm{ov}(A,L^*)\,\mathbb{C}\mathrm{ov}(A,AL^*)}{\mathbb{V}\mathrm{ar}(L^*)\,\mathbb{V}\mathrm{ar}(A) - \mathbb{C}\mathrm{ov}(A,L^*)^2}, \\
u_0 &= \overline{AL^*} - u_A\overline{A} - u_{L^*}\overline{L^*},
\end{aligned}
$$

where $\overline{AL^*}, \overline{A}$, and $\overline{L^*}$ denote the mean of A times L^*, A, and L^*, respectively.

If we want to express u_A and u_{L^*} in terms of λ, π_0, π_1, p_0, and p_1, we can write a linear model for A conditional on L^* denoting that $P(A=1|L^*=l^*) = \pi^*_{l^*}$ and using standard regression theory to get an expression for $\mathbb{C}\mathrm{ov}(A,L^*)$:

$$
\mathbb{E}[A|L^*] = \pi^*_0 + (\pi^*_1 - \pi^*_0)L^*, \quad \pi^*_1 - \pi^*_0 = \frac{\mathbb{C}\mathrm{ov}(A,L^*)}{\mathbb{V}\mathrm{ar}(L^*)}, \quad \text{thus} \quad \mathbb{C}\mathrm{ov}(A,L^*) = (\pi^*_1 - \pi^*_0)\,\mathbb{V}\mathrm{ar}(L^*),
$$

where $\mathbb{V}\mathrm{ar}(L^*) = \ell(1-\ell)$. Since $\mathbb{E}[AL^*|L^*=0] = 0$ and $\mathbb{E}[AL^*|L^*=1] = \mathbb{E}[A|L^*=1] = \pi^*_1$, it follows,

$$
\mathbb{E}[AL^*|L^*] = \pi^*_1 L^*, \quad \pi^*_1 = \frac{\mathbb{C}\mathrm{ov}(AL^*,L^*)}{\mathbb{V}\mathrm{ar}(L^*)}, \quad \text{thus} \quad \mathbb{C}\mathrm{ov}(AL^*,L^*) = \pi^*_1\,\mathbb{V}\mathrm{ar}(L^*).
$$

Equivalently, since $\mathbb{E}[AL^*|A=0] = 0$ and $\mathbb{E}[AL^*|A=1] = \mathbb{E}[L^*|A=1]$, it follows that,

$$
\mathbb{E}[AL^*|A] = \mathbb{E}[L^*|A=1]A = \frac{P(A=1|L^*=1)P(L^*=1)}{P(A=1)}A,
$$

$$\mathbb{E}[L^*|A=1] \;=\; \frac{\pi_1^*\ell}{\omega}, \quad \frac{\pi_1^*\ell}{a} = \frac{\mathbb{C}\mathrm{ov}(AL^*, A)}{\mathbb{V}\mathrm{ar}(A)}, \quad \text{thus} \quad \mathbb{C}\mathrm{ov}(AL^*, A) = \frac{\pi_1^*\ell}{\omega}\,\mathbb{V}\mathrm{ar}(A).$$

Here, $\mathbb{V}\mathrm{ar}(A) = \omega(1-\omega)$, and $\mathbb{V}\mathrm{ar}(L^*) = \ell(1-\ell)$. Denoting that $\omega = P(A=1) = \pi_0^*(1-\ell) + \pi_1^*\ell$. Combining the different expressions gives,

$$\begin{aligned}
u_A &= \frac{\pi_1^*\ell/\omega\,\mathbb{V}\mathrm{ar}(A)\,\mathbb{V}\mathrm{ar}(L^*) - \pi_1^*(\pi_1^* - \pi_0^*)\,\mathbb{V}\mathrm{ar}(L^*)^2}{\mathbb{V}\mathrm{ar}(A)\,\mathbb{V}\mathrm{ar}(L^*) - (\pi_1 - \pi_0)^2\,\mathbb{V}\mathrm{ar}(L^*)^2} \\
&= \frac{\pi_1^*\ell/\omega\,\mathbb{V}\mathrm{ar}(A) - \pi_1^*(\pi_1^* - \pi_0^*)\,\mathbb{V}\mathrm{ar}(L^*)}{\mathbb{V}\mathrm{ar}(A) - (\pi_1 - \pi_0)^2\,\mathbb{V}\mathrm{ar}(L^*)} \\
&= \ell \times \frac{\pi_1^*(1-\omega) - \pi_1^*(\pi_1^* - \pi_0^*)(1-\ell)}{\omega(1-\omega) - (\pi_1^* - \pi_0^*)^2\ell(1-\ell)} \\
&= \ell \times \frac{\pi_1^* - {\pi_1^*}^2}{(\pi_1^* - {\pi_1^*}^2)\ell + (\pi_0^* - {\pi_0^*}^2)(1-\ell)}, \\
u_{L^*} &= \frac{\pi_1^*\,\mathbb{V}\mathrm{ar}(A)\,\mathbb{V}\mathrm{ar}(L^*) - \pi_1^*\ell/\omega(\pi_1^* - \pi_0^*)\,\mathbb{V}\mathrm{ar}(A)\,\mathbb{V}\mathrm{ar}(L^*)}{\mathbb{V}\mathrm{ar}(L^*)\,\mathbb{V}\mathrm{ar}(A) - ((\pi_1^* - \pi_0^*)\,\mathbb{V}\mathrm{ar}(L^*))^2} \\
&= \frac{\pi_1^*\omega - \pi_1^*\ell(\pi_1^* - \pi_0^*)}{\omega - (\pi_1^* - \pi_0^*)^2\,\mathbb{V}\mathrm{ar}(L^*)/(1-\omega)} \\
&= \frac{\pi_1^*\pi_0^*(1 - {\pi_1^*}^2)\ell + \pi_1^*\pi_0^*(1 - {\pi_0^*}^2)(1-\ell)}{(\pi_1^* - {\pi_1^*}^2)\ell + (\pi_0^* - {\pi_0^*}^2)(1-\ell)} \\
u_0 &= \overline{AL^*} - u_A\overline{A} - u_{L^*}\overline{L^*}.
\end{aligned}$$

The intercept, the coefficient for A and the coefficient for L^* of the conditional regression model for Y given A and L^* which includes only main effects of A and L^* are, respectively:

$$\begin{aligned}
&\alpha + \gamma\phi_{00} + \delta u_0, \\
\beta + \gamma(\phi_{10} - \phi_{00})\Big(1 - \ell \times \{&\frac{\pi_1^* - {\pi_1^*}^2}{(\pi_1^* - {\pi_1^*}^2)\ell + (\pi_0^* - {\pi_0^*}^2)(1-\ell)}\}\Big) \\
+\gamma(\phi_{11} - \phi_{01})\Big(\ell \times \{&\frac{\pi_1^* - {\pi_1^*}^2}{(\pi_1^* - {\pi_1^*}^2)\ell + (\pi_0^* - {\pi_0^*}^2)(1-\ell)}\}\Big), \\
&\text{and} \quad \gamma(\phi_{01} - \phi_{00}) + \delta u_{L^*}.
\end{aligned}$$

2 Marginal structural model estimated using inverse probability weighting

Under the assumptions described in section of the main article, an MSM-IPW under model (2) is estimated by fitting a linear regression model for A on Y, where each subject i is weighted by 1 over the probability of that subject's observed exposure given the misclassified confounding variable L^*. Hence, an MSM-IPW proceeds by solving the weighted regression

model,

$$\sum_{i=1}^{n} \frac{1}{P(A_i|L_i^*)}(Y_i - \alpha_{\text{msm}} - \beta A_i) = 0 \quad \text{and} \quad \sum_{i=1}^{n} \frac{A_i}{P(A_i|L_i^*)}(Y_i - \alpha_{\text{msm}} - \beta A_i) = 0.$$

Solving these equations for α_{msm} and β result in the following estimators:

$$\hat{\alpha}_{\text{msm}} = \overline{Y}_{w^*} - \hat{\beta}_{\text{msm}}\overline{A}_{w^*} \quad \text{and} \quad \hat{\beta} = \frac{\sum_{i=1}^{n} \frac{1}{P(A_i|L_i)}(Y_i - \overline{Y}_{w^*})(A_i - \overline{A}_{w^*})}{\sum_{i=1}^{n} \frac{1}{P(A_i|L_i)}(A_i - \overline{A}_{w^*})^2},$$

where,

$$\overline{Y}_{w^*} = \frac{\sum_{i=1}^{n} Y_i/P(A_i|L_i^*)}{\sum_{i=1}^{n} 1/P(A_i|L_i^*)} \quad \text{and} \quad \overline{A}_{w^*} = \frac{\sum_{i=1}^{n} A_i/P(A_i|L_i^*)}{\sum_{i=1}^{n} 1/P(A_i|L_i^*)}.$$

Let n_{al}^* be the number of subjects with $A = a$ and $L^* = l^*$ and n_{al} be the number of subjects with $A = a$ and $L = l$. In a population of n subjects,

$$\begin{aligned}
n_{00}^* &= nP(A = 0, L^* = 0) = nP(A = 0|L^* = 0)P(L^* = 0) \\
&= n\sum^{l} P(A = 0|L = l, L^* = 0)P(L = l|L^* = 0)P(L^* = 0) \\
&= n\sum^{l} P(A = 0|L = l)P(L = l|L^* = 0)P(L^* = 0) \\
&= n\sum^{l} P(A = 0|L = l)P(L = l)P(L^* = 0|L = l) \\
&= n_{00}(1 - p_0) + n_{01}(1 - p_1),
\end{aligned}$$

which relies on the assumption that L^* is non-differentially misclassified with respect tot the exposure (i.e., $L^* \perp\!\!\!\perp A|L$). Equivallently,

$$n_{01}^* = n_{00}p_0 + n_{01}p_1, \quad n_{10}^* = n_{10}(1 - p_0) + n_{11}(1 - p_1) \quad \text{and} \quad n_{11}^* = n_{10}p_0 + n_{11}p_1.$$

Hence,

$$\begin{aligned}
\sum_{i=1}^{n} 1/P(A_i|L_i^*) &= \sum_{i=1}^{n} \frac{1}{\sum_l [P(A_i|L_i^*, L = l)P(L = l|L_i^*)]} \\
&= \sum_{i=1}^{n} \frac{1}{\sum_l [P(A_i|L = l)P(L = l|L_i^*)]} \\
&= \sum^{n_{00}^*} \frac{1}{\sum_l [(1 - \pi_l)P(L = l|L^* = 0)]} + \sum^{n_{01}^*} \frac{1}{\sum_l [(1 - \pi_l)P(L = l|L^* = 1)]}
\end{aligned}$$

$$+ \sum^{n^*_{10}} \frac{1}{\sum_l [\pi_l P(L=l|L^*=0)]} + \sum^{n^*_{11}} \frac{1}{\sum_l [\pi_l P(L=l|L^*=1)]}.$$

Here,

$$\begin{aligned}
\sum^{n^*_{00}} \frac{1}{\sum_l [(1-\pi_l) P(L=l|L^*=0)]} &= \frac{n_{00}(1-p_0)+n_{01}(1-p_1)}{(1-\pi_0)P(L=0|L^*=0)+(1-\pi_1)P(L=1|L^*=0)} = \\
&= \frac{n_{00}(1-p_0)+n_{01}(1-p_1)}{(1-\pi_0)\frac{P(L^*=0|L=0)(1-\lambda)}{P(L^*=0)}+(1-\pi_1)\frac{P(L^*=0|L=1)\lambda}{P(L^*=0)}} = \\
&= \frac{n_{00}(1-p_0)+n_{01}(1-p_1)}{\frac{n_{00}}{nP(L^*=0)}(1-p_0)+\frac{n_{01}}{nP(L^*=0)}(1-p_1)} \\
&= \frac{1}{1/(nP(L^*=0))} \\
&= nP(L^*=0) = n(1-\ell), \\
\sum^{n^*_{01}} \frac{1}{\sum_l [(1-\pi_l) P(L=l|L^*=1)]} &= nP(L^*=1) = n\ell, \\
\sum^{n^*_{10}} \frac{1}{\sum_l [\pi_l P(L=l|L^*=0)]} &= nP(L^*=0) = n(1-\ell), \\
\sum^{n^*_{11}} \frac{1}{\sum_l [\pi_l P(L=l|L^*=1)]} &= nP(L^*=1) = n\ell.
\end{aligned}$$

From these expressions it follows that,

$$\sum_{i=1}^{n} 1/P(A_i|L^*_i) = 2n(1-\ell) + 2n\ell = 2n.$$

Further,

$$\begin{aligned}
\sum_{i=1}^{n} \mathbb{E}[Y_i]/P(A_i|L^*_i) &= \sum^{n^*_{00}} \frac{\mathbb{E}[Y_i]}{\sum_l [(1-\pi_l) P(L=l|L^*=0)]} + \sum^{n^*_{01}} \frac{\mathbb{E}[Y_i]}{\sum_l [(1-\pi_l) P(L=l|L^*=1)]} \\
&+ \sum^{n^*_{10}} \frac{\mathbb{E}[Y_i]}{\sum_l [\pi_l P(L=l|L^*=0)]} + \sum^{n^*_{11}} \frac{\mathbb{E}[Y_i]}{\sum_l [\pi_l P(L=l|L^*=1)]} \\
&= \sum^{n^*_{00}} \frac{\alpha+\gamma P(L=1|A=0,L^*=0)}{\sum_l [(1-\pi_l) P(L=l|L^*=0)]} + \sum^{n^*_{01}} \frac{\alpha+\gamma P(L=1|A=0,L^*=1)}{\sum_l [(1-\pi_l) P(L=l|L^*=1)]} \\
&+ \sum^{n^*_{10}} \frac{\alpha+\beta+\gamma P(L=1|A=1,L^*=0)}{\sum_l [\pi_l P(L=l|L^*=0)]} + \sum^{n^*_{11}} \frac{\alpha+\beta+\gamma P(L=1|A=1,L^*=1)}{\sum_l [\pi_l P(L=l|L^*=1)]}
\end{aligned}$$

$$\begin{aligned}
&= n\alpha(1-\ell) + n\gamma(1-\ell)\phi_{00} + n\alpha\ell + n\gamma\phi_{01} + n(\alpha+\beta)(1-\ell) + n\gamma(1-\ell)\phi_{10} \\
&+ n(\alpha+\beta)\ell + n\gamma\phi_{11} \\
&= 2n\alpha + n\beta + n\gamma(1-\ell)(\phi_{00}+\phi_{10}) + n\gamma\ell(\phi_{01}+\phi_{11}),
\end{aligned}$$

and,

$$\begin{aligned}
\sum_{i=1}^{n} A_i/P(A_i|L_i) &= \sum^{n^*_{10}} \frac{1}{\sum_l [\pi_l P(L=l|L^*=0)]} + \sum^{n^*_{11}} \frac{1}{\sum_l [\pi_l P(L=l|L^*=1)]} \\
&= n(1-p_0)(1-\lambda) + n(1-p_1)\lambda + np_0(1-\lambda) + np_1\lambda = n.
\end{aligned}$$

Combining these expressions leads to,

$$\mathbb{E}[\overline{Y}_{w^*}] = \alpha + \beta/2 + \gamma/2(1-\ell)(\phi_{00}+\phi_{10}) + \gamma/2\ell(\phi_{01}+\phi_{11}) \quad \text{and} \quad \overline{A}_{w^*} = n/2n = 1/2,$$

and,

$$\begin{aligned}
\sum_{i=1}^{n} \frac{(A_i - \overline{A}_{w^*})^2}{P(A_i|L_i^*)} &= \sum^{n^*_{00}} \frac{(-1/2)^2}{\sum_l [(1-\pi_l)P(L=l|L^*=0)]} \\
&+ \sum^{n^*_{01}} \frac{(-1/2)^2}{\sum_l [(1-\pi_l)P(L=l|L^*=1)]} \\
&+ \sum^{n^*_{10}} \frac{(1-1/2)^2}{\sum_l [\pi_l P(L=l|L^*=0)]} \\
&+ \sum^{n^*_{11}} \frac{(1-1/2)^2}{\sum_l [\pi_l P(L=l|L^*=1)]} \\
&= 1/4 \times \sum_{i=1}^{n} 1/P(A_i|L_i^*) = n/2.
\end{aligned}$$

$$\begin{aligned}
\sum_{i=1}^{n} \frac{\mathbb{E}[(Y_i - \overline{Y}_{w^*})](A_i - \overline{A}_{\tilde{w}})}{P(A_i|L_i^*)} &= \sum^{n^*_{00}} \frac{\beta/4 - \gamma/2\phi_{00} + \gamma/4(1-\ell)(\phi_{00}+\phi_{10}) + \gamma/4\ell(\phi_{01}+\phi_{11})}{\sum_l [(1-\pi_l)P(L=l|L^*=0)]} \\
&+ \sum^{n^*_{01}} \frac{\beta/4 - \gamma/2\phi_{01} + \gamma/4(1-\ell)(\phi_{00}+\phi_{10}) + \gamma/4\ell(\phi_{01}+\phi_{11})}{\sum_l [(1-\pi_l)P(L=l|L^*=1)]} \\
&+ \sum^{n^*_{10}} \frac{\beta/4 + \gamma/2\phi_{10} - \gamma/4(1-\ell)(\phi_{00}+\phi_{10}) - \gamma/4\ell(\phi_{01}+\phi_{11})}{\sum_l [\pi_l P(L=l|L^*=0)]} \\
&+ \sum^{n^*_{11}} \frac{\beta/4 + \gamma/2\phi_{11} - \gamma/4(1-\ell)(\phi_{00}+\phi_{10}) - \gamma/4\ell(\phi_{01}+\phi_{11})}{\sum_l [\pi_l P(L=l|L^*=0)]} \\
&= n(1-\ell)(\beta/4 - \gamma/2\phi_{00} + \gamma/4(1-\ell)(\phi_{00}+\phi_{10}) + \gamma/4\ell(\phi_{01}+\phi_{11})) \\
&+ n\ell(\beta/4 - \gamma/2\phi_{01} + \gamma/4(1-\ell)(\phi_{00}+\phi_{10}) + \gamma/4\ell(\phi_{01}+\phi_{11}))
\end{aligned}$$

$$
\begin{aligned}
&+ \quad n(1-\ell)(\beta/4 + \gamma/2\phi_{10} - \gamma/4(1-\ell)(\phi_{00}+\phi_{10}) - \gamma/4\ell(\phi_{01}+\phi_{11})) \\
&+ \quad n\ell(\beta/4 + \gamma/2\phi_{11} - \gamma/4(1-\ell)(\phi_{00}+\phi_{10}) - \gamma/4\ell(\phi_{01}+\phi_{11})) \\
&= \quad n/2(\beta(1-\ell) + \beta\ell - \gamma(1-\ell)\phi_{00} - \gamma\ell\phi_{01} + \gamma(1-\ell)\phi_{10} + \gamma\ell\phi_{11}) \\
&= \quad n/2(\beta + \gamma(1-\ell)(\phi_{10}-\phi_{00}) + \gamma\ell(\phi_{11}-\phi_{01}).
\end{aligned}
$$

The above mentioned leads to the following expression for the expected estimated value of the effect of A, based on the MSM-IPW,

$$
\mathbb{E}[\hat{\beta}] = \beta + \gamma(\phi_{10}-\phi_{00})(1-\ell) + \gamma(\phi_{11}-\phi_{01})\ell \quad \text{and} \quad
\begin{aligned}
\mathbb{E}[\hat{\alpha}_{\text{msm}}] &= \alpha + \gamma/2 \times [2(1-\ell)\phi_{00} + 2\ell\phi_{01})] \\
&= \alpha + \gamma\phi_{00}(1-\ell) + \gamma\phi_{01}\ell.
\end{aligned}
$$

eAppendix2 Illustration: quantitative bias analysis of classification error in a confounding variable

Using an example study of blood pressure lowering therapy, we illustrate how the bias expressions in section of the main article can be used to perform a quantitative bias analysis for misclassification of a confounding variable. For our illustration we use data of the National Health And Nutritional Examination Survey (NHANES) [36, 37]. Specifically, we study the average treatment effect of diuretic use ($A = 1$) in comparison to beta blocker use ($A = 0$) on systolic blood pressure (Y) using two approaches: by inverse weighting with the propensity for diuretic or beta blocker use given self-reported categorical body mass index (BMI) (L^*), and using a conditional linear regression with adjustment for self-reported categorical BMI. This supplement comprises background material that complements the motivating example in the main article. Additionally, equations are derived to inform the quantitative bias analysis.

NHANES. The NHANES survey consists of questionnaires, followed by a standardized health examination in specially equipped mobile examination centers. In the 2011-2014 sample 19,151 participants were physically examined. Of the 19,151 physically examined people, 12,185 participants aged over 16 were asked to fill out a questionnaire, including questions on self-reported weight and height, used to calculate self-reported BMI. For this illustration, we used complete data on 585 users of diuretics and 824 users of beta blockers (excluding non-users and people using both).

Parameters estimated in NHANES. In the NHANES data, it was found that the prevalence of self-reported overweight/obese was 0.77 (ℓ), the probability of receiving treatment given that one self-reports to be underweight/normal weight is 0.32 (π^*_0), the probability of receiving treatment given that one self-reports to be overweight/obese is 0.44 (π^*_1). Finally, the association between L^* and Y, given that $A = 0$ estimated in a conditional regression model including an interaction between A and L* was -6.63.

BMI measured by trained technicians. In the NHANES, anthropometric measures were also taken by trained health technicians. By using these measures to calculate BMI category, we found that the specificity of self-reported BMI category was 0.94 (p_1), and the sensitivity was 0.92 ($p_0 = 0.08$). The average treatment effect (95 % CI) of diuretics use in comparison to beta blocker use on mean blood pressure was -3.59 (-5.84; -1.35) estimated using MSM-IPW (by inverse weighting with the propensity for diuretic or beta blocker use given categorical BMI). Given that a subject is not overweight/obese, the fitted weights were 1.48 and 3.09 for beta blocker and diuretics use, respectively. Given that a subject is overweight/obese, the fitted weights were 1.77 and 2.30, respectively. In comparison, if self-reported categorical BMI was used, the fitted weights slightly differed: 1.46, 3.17, 1.79 and 2.26, respectively. Consequently, estimates of the average treatment effect differed, depending on the BMI measure that was used to calculate the inverse probability weights (-3.59 using categorical BMI versus -3.52 using categorical self-reported BMI (Table 3, main article)).

Performing a quantitative bias analysis. To inform a quantitative bias analysis, one needs to specify the bias parameters for sensitivity (p_1) and specificity ($1 - p_0$) using

external validation data, internal validation data, or an educated guess. From the data, one can estimate the prevalence of misclassified confounding variable L^* (i.e., ℓ), the probability of receiving treatment given that L^* is null (i.e., π_0^*) and the probability of receiving treatment given that L^* is one (i.e., π_1^*). We calculate the probability of receiving treatment given that L is null or one (i..e, π_0, and π_1, respectively) using the data and the assumed values of p_0 and p_1. Since,

$$\pi_0^* = \frac{\pi_0(1-p_0)(1-\lambda) + \pi_1(1-p_1)\lambda}{(1-\ell)}, \quad \text{and} \quad \pi_1^* = \frac{\pi_0 p_0(1-\lambda) + \pi_1 p_1 \lambda}{\ell},$$

it follows that if $p_0 = 1$, $\pi_1 = \pi_0^*$ and if $p_1 = 0$, $\pi_0 = \pi_1^*$ (using that $0 < \ell < 1$, as used in eAppendix1 section 1). Further, if $p_0 = 1$ and $0 < p_1 < 1$, we obtain,

$$\pi_1 = \pi_0^*, \quad \text{and} \quad \pi_0 = \frac{\pi_0^* p_1 \lambda - \pi_1^* \ell}{(1-\lambda)}.$$

Additionally, if $p_1 = 0$ and $0 < p_0 < 1$, we obtain

$$\pi_0 = \pi_1^*, \quad \text{and} \quad \pi_1 = \frac{\pi_0^*(1-\ell) - \pi_1^*(1-p_0)(1-\lambda)}{\lambda}.$$

If we assume that $p_0 \neq 1$ and $p_1 \neq 0$ and use our assumption that $0 < \lambda < 1$, it follows that,

$$\pi_0 = \frac{\pi_0^*(1-\ell) - \pi_1(1-p_1)\lambda}{(1-p_0)(1-\lambda)}, \quad \pi_1 = \frac{\pi_1^* \ell - \pi_0 p_0 (1-\lambda)}{p_1 \lambda}. \tag{eA2.1}$$

By rewriting the expression for π_1 using the expression for π_0, it follows that,

$$\begin{aligned}
\pi_1 &= \frac{\pi_1^* \ell - \pi_0 p_0(1-\lambda)}{p_1 \lambda} \\
&= \frac{\pi_1^* \ell - \frac{\pi_0^*(1-\ell) - \pi_1(1-p_1)\lambda}{(1-p_0)(1-\lambda)} p_0 (1-\lambda)}{p_1 \lambda} \\
&= \frac{\pi_1^* \ell - (\pi_0^*(1-\ell) - \pi_1(1-p_1)\lambda)\frac{p_0}{(1-p_0)}}{p_1 \lambda} \\
&= \frac{\pi_1^* \ell - \pi_0^*(1-\ell)\frac{p_0}{(1-p_0)} + \frac{(1-p_1)p_0}{(1-p_0)}\lambda \pi_1}{p_1 \lambda} \\
&= \frac{\pi_1^* \ell - \pi_0^*(1-\ell)\frac{p_0}{(1-p_0)}}{p_1 \lambda} + \frac{(1-p_1)p_0}{(1-p_0)p_1}\pi_1 \\
&= \frac{\pi_1^* \ell - \pi_0^*(1-\ell)\frac{p_0}{(1-p_0)}}{p_1 \lambda} + \frac{(1-p_1)p_0}{(1-p_0)p_1}\pi_1.
\end{aligned}$$

Consequently,

$$
\begin{aligned}
(1-\frac{(1-p_1)p_0}{(1-p_0)p_1})\pi_1 &= \frac{\pi_1^*\ell-\pi_0^*(1-\ell)\frac{p_0}{(1-p_0)}}{p_1\lambda}, \\
\pi_1 &= \frac{\frac{\pi_1^*\ell-\pi_0^*(1-\ell)\frac{p_0}{(1-p_0)}}{p_1\lambda}}{\frac{(1-p_0)p_1-(1-p_1)p_0}{(1-p_0)p_1}} \\
&= \frac{\pi_1^*\ell-\pi_0^*(1-\ell)\frac{p_0}{(1-p_0)}}{p_1\lambda}\times\frac{(1-p_0)p_1}{(1-p_0)p_1-(1-p_1)p_0}. \qquad \text{(eA2.2)}
\end{aligned}
$$

From expression (eA2.2) we now obtain a value for π_1, which we use to obtain a value for π_0 from expression (eA2.1). We calculate the prevalence of L (i.e., λ) by,

$$
\lambda = p_0, \quad \text{if } p_0 = p_1 \quad \text{and} \quad \lambda = \frac{\ell - p_0}{p_1 - p_0} \quad \text{otherwise.}
$$

Subsequently, the expressions for π_0, π_1 and λ can be used to obtain estimates for ϕ_{al^*} using the expression in eAppendix1, section 1. Lastly, an estimate for γ can be obtained by fitting a conditional regression model on Y given A and L^*, including an interaction between A and L^*. The coefficient for L^* from this model is then divided by $(\phi_{01} - \phi_{00})$ to get an estimate for γ, holding that $\phi_{01} \neq \phi_{00}$. The inequality $\phi_{01} \neq \phi_{00}$ holds if $p_0 \neq p_1$, in the case that $p_0 = p_1$, γ is not identifiable from the data (and thus, bias is not identifiable). The bias expressions (3) and (4) in the main text of the article can subsequently be used to calculate bias in the average treatment effect estimator.
